# Large Congenital Pericardial Cyst Presented by Palpitation and Left Ventricle Posterior Wall Compression: A Rare Case Report

**DOI:** 10.3390/pediatric13010007

**Published:** 2021-01-15

**Authors:** Noor Mohamad Noori, Elham Shafighi Shahri, Seyed Hosein Soleimanzadeh Mousavi

**Affiliations:** Children and Adolescents Health Research Center, Ali-Ibn-Abitaleb Hospital, Zahedan University of Medical Sciences, Zahedan 9816743463, Iran; dr_noori_cardio@yahoo.com (N.M.N.); elham.shafighi2276@gmail.com (E.S.S.)

**Keywords:** pericardial cyst, mediastinal cyst, cardiac surgery, pediatrics

## Abstract

Congenital pericardial cysts are rare anomalies caused by the failure of fetal lacunae to coalesce into pericardial coelom. In this article a 9-year-old boy admitted with complain of palpitation in daily activities. The electrocardiography detected sinus tachycardia of 150 beats per minute with normal axis. Although chest X ray were normal, echocardiography showed an abnormal mass that compressed the posterior wall of left ventricle. The mass was extrinsic and confined to the pericardium. After midsternotomy, a huge cyst was found and totally excised. The complications of pericardial cyst can be significant, and the diagnosis relies on a careful examination and radiographic findings.

## 1. Introduction

Congenital pericardial cysts are rare anomalies caused by the failure of fetal lacunae to coalesce into the pericardial coelom. Pericardial cysts were recognized as a congenital malformation and diverticulum in the 1940s. The incidence rate of pericardial cysts, which was reported by Le Roux in 1958, is one in every 100,000 individuals [1,2,3,4,5,6,7,8].

Pericardial cysts have been found in 6–7% of mediastinal mass overall. They are usually observed in the third and fourth decades of life, and the occurrence rate in both genders is equal [1,2,4].

Pericardial cysts are generally unilocular, thin-walled, and wrinkled by endothelium or mesothelium, and they contain translucent fluid [1,2]. Regarding location, 70% of cases are located in the right cardiophrenic angle, 22% are in the left cardiophrenic angle, and 8% are located in the posterior or the anterior-superior part of the mediastinum [2,7,8].

More than 50% of pericardial cysts are asymptomatic and discovered incidentally, despite others having various symptoms at presentation [3]. Dyspnea, chest pain, persistent cough, hemoptysis, fever, and pneumothorax are rare symptoms, and are classified as an emergency if happening [9]. They occasionally present with symptoms related to compression of adjacent structures.

In this case report, we describe a 9-year-old child with a pericardial cyst by left ventricle posterior wall compression with unusual symptoms of palpitations during routine activity. Giant pericardial cysts are rare, and available published articles in the literature are few. With improvement in technology and computed tomography (CT) scans we were able to diagnose this benign congenital anomaly before surgery.

## 2. Case Presentation

A 9-year-old boy presented to the emergency department with palpitations in daily activities, and without any other complications and no past medical history detected. He had such episodes before and was treated by beta blockers (propranolol) without any recuperation during the past year. The patient was clinically stable. His blood pressure was 100/60 mm Hg at the initial, and his pulse rate was 130 beats per minute. In auscultation of both lungs found nothing and abdominal, spleen and liver examination, were normal. 

**Figure 1:**
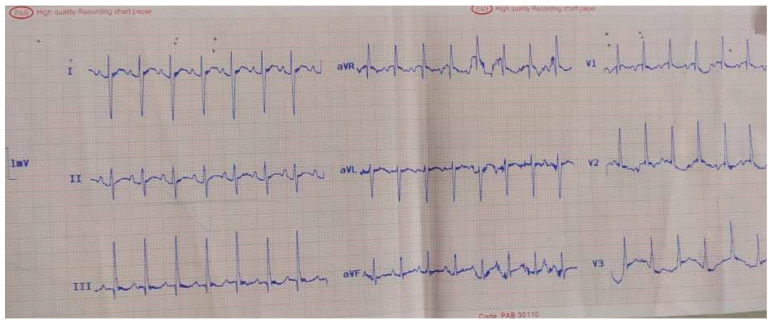
A 9-year-old boy presented with palpitations. Electrocardiography showed sinus tachycardia.

**Figure 2:**
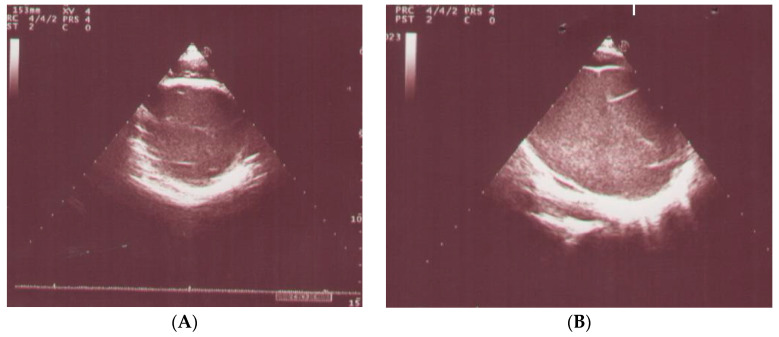

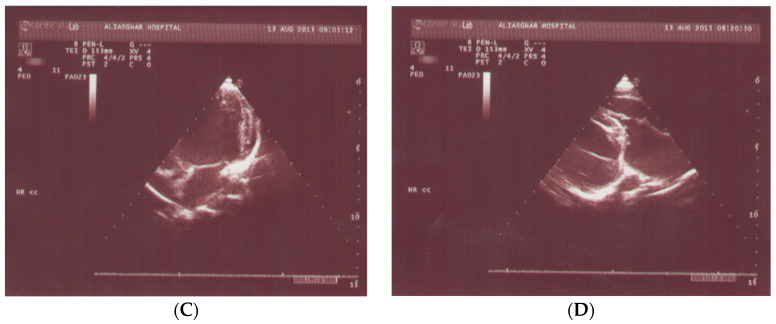
Echocardiography showed an abnormal mass in the posterior wall of the left ventricle. (**A**) Parasternal Short Axis view. (**B**) Left ventricle pericardial cyst. (**C**) Parasternal Long Axis view. (**D**) Apical 4-chamber view.

**Figure 3:**
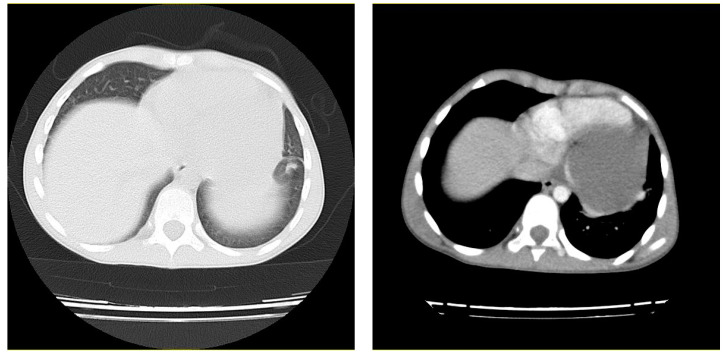
Axial view of the patient’s contrast-enhanced chest computed tomography (CT) scan. A retrosternal mass characterized with a clear serous fluid with compression on the posterior wall of the left ventricle.

The electrocardiography (ECG) detected sinus tachycardia with normal axis (Figure 1). Although plane chest radiography was normal, an apical four-chamber view echocardiography showed an echolucent space next to the posterior wall of the left ventricle with compression effect (Figure 2). The mass was extrinsic and confined to the pericardium. The right and left ventricular chamber size and function were normal, and there was no valvular heart disease. Then, a chest CT scan with contrast showed a retrosternal mass characterized with a clear serous fluid with compression on the posterior wall of the left ventricle (Figure 3). To rule out the causes, inflammatory factors, blood culture, viral antibodies, collagen vascular markers, sputum acid-fast bacilli (AFB) and PPD test results all were normal.

**Figure 4:**
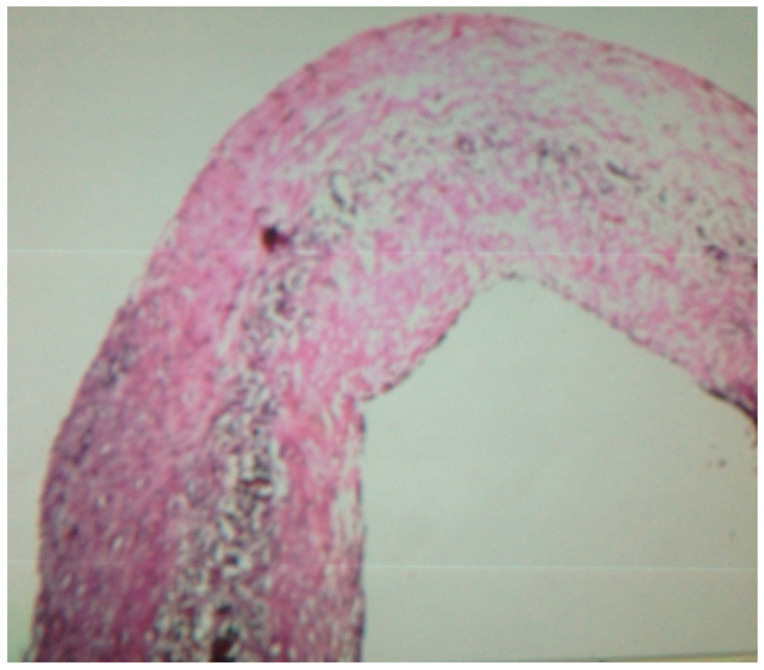
Low-power view of pericardial cyst showing the mesothelial lining and cyst wall composed of loose connective tissue with blood vessels.

**Figure 5:**
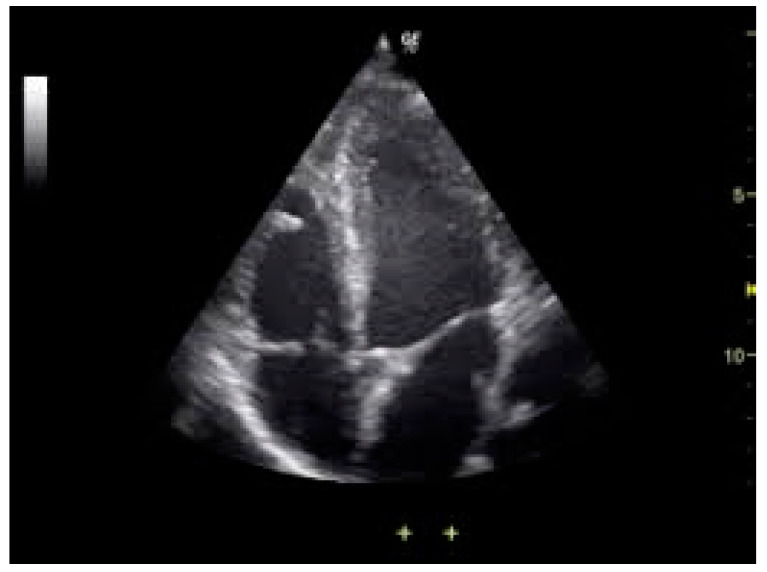
Postoperative echocardiography showed normal findings.

The patient was referred to a cardiac surgeon. Surgeon accessed to the mediastinum after median sternotomy, then the pericardium opened, and then an 8×7×6 cm anterior mediastinal cyst filled with necrotic debris was found. The mass was removed and was sent to a pathologist for further evaluation, and it was reported as a pericardial cyst with mesothelial lining and a cyst wall composed of loose connective tissue (Figure 4). The cyst had thick walls and compressed the left ventricle. After 10 days, the patient was discharged and a 12-month follow-up by transthoracic echocardiogram did not demonstrate any mass in the pericardium (Figure 5). The patient remains symptom-free after 12 months.

## 3. Discussion

Pericardial cysts are rare and benign, measuring commonly from 1 to 5 cm in diameter. Pericardial cysts with a diameter above 10 cm are extremely rare and are defined as Giant Pericardial Cysts [3,8]. They largely are asymptomatic. Although several patients with giant pericardial cysts might be asymptomatic, some cases reveal life-threatening complications [1,2,3,4,5,6,7,8]. Common symptoms that are acquired more by mediastinal compression are dyspnea, chest pain and dry cough—more reported in patients with giant pericardial cysts [2,3]. A prominent pericardial fat pad, ventricular aneurysms, pericardial hematoma, and mediastinal mass are mostly confused with cysts at the time of diagnosis [2,3].

This case is a unique presentation of a patient who presented with palpitations at the emergency department. Except for a history of propranolol use, the patient had no history of medical examination for symptoms. His presentation of palpitations is a common normal variation in young children and adolescents in school-aged groups. This child was suffering from them, and they affected routine activities. At first, all the laboratory data and routine paraclinical findings were normal and made for confusion about an absolute diagnosis. To rule out a cardiac disorder, cardiologic consultation was done; after echocardiography, a pericardial cyst was contemplated as a differential diagnosis. After a contrast-enhanced chest CT scan, the pericardial cyst as a definite diagnosis was made.

These cysts have varied from mild to life-threating complications [3]. Some complications reported are cyst rupture, erosion of the cyst into near structures, right ventricular wall compression, superior vena cava compression, cardiac tamponade, mitral valve prolapses, obstruction of the right main bronchus, atrial fibrillation, and even sudden death [2,8].

To evaluate and detect pericardial cysts, radiographic examination (plane chest radiograph), echocardiography, computerized tomography, and magnetic resonance imaging are most frequently used [7,10,11]. It is noted that the results of echocardiography must be confirmed by computed tomography and magnetic resonance imaging [2,7,8,12,13]. Nevertheless, differentiating malignancies from nonmalignant fluid-filled cysts in patients with a pericardial anomaly is actually a task of great difficulty and, therefore, requires serious precision [2,11].

Two-dimensional echocardiography was first used by Hynes et al. for the diagnosis of pericardial cysts [7]. This modality was also used for a differential diagnosis of pericardial cysts from a prominent fat pad, a left ventricular aneurysm, a prominent left atrial appendage, an aortic aneurysm, and solid tumors [7]. Color and Doppler echocardiography are also used for differential diagnosis of pericardial cysts from other vascular abnormalities, such as coronary aneurysms [7,8]. If transthoracic echocardiography is insufficient in the diagnosis of pericardial cysts, transesophageal echocardiography can be beneficial in providing precise diagnosis, and differentiating them from other conditions [8].

Pericardial cysts originate mostly congenitally. To describe the origination of these cysts, some lectures were reviewed, and the results are listed by prevalence, respectively, in Table 1. Adrian Lambert and Mazer described the anatomy and cyst formation steps directly [14,15]. The showed that cysts can establish from the disconnection of mesenchymal lacunae. Then the fluid shifts from pericardial diverticula to the pericardial sac. On the other hand, Lillie et al. explained the formation of cysts by the concept of differential persistence and graded constriction of ventral recess of the pericardial coelom, results to diverticulum with a narrow neck or results in the origin of a pericardial cyst in communication with the pericardial cavity [16]. In addition, some cases were reported as spontaneous regression of pericardial cysts [17].

Prenatal diagnosis of a pericardial cyst is made possible with ultrasound examination beyond the 14th week of gestation [14].

Most medical experts recommend surgical excision of pericardial cysts in symptomatic patients; however, asymptomatic cases with pericardial cysts are managed conventionally with a close follow-up. In addition, symptomatic pericardial cysts have also been successfully removed through video-assisted thoracic surgery [1,5,8,10]. Since thoracoscopy resection of pericardial cysts reduces surgical trauma and postoperative pain, shortens the recovery period, and provides more medically desirable outcomes, it is quite a good alternative decision instead of open surgical resection. Percutaneous aspiration of cyst contents is another alternative method to surgical resection of pericardial cysts in symptomatic patients [5,8,12].

Onur Omaygenc et al. (2016) reported a huge pericardial cyst located at the right cardio phrenic angle impairing ventricular filling properties, without causing evident symptoms. The cyst was almost 13 cm in the largest diameter. Echocardiography and computed tomography scan were utilized not only to confirm the diagnosis, but also to determine the treatment strategy. They introduced surgical or percutaneous interventional treatment as the best options. However, pericardial cysts might be occasionally life-threating, depending on the location of the cyst and its relationship with the adjacent structures [19].

Alkharabsheh et al. (2016) conducted a retrospective observational study of all patients diagnosed with pericardial cysts between 2008–2014 based on characteristic imaging findings on chest computed tomography and/or cardiac magnetic resonance imaging. They concluded that a majority of patients suffering from pericardial cyst are asymptomatic. Interval enlargement of the pericardial cysts is infrequent, and it is unlikely to be clinically relevant. Additionally, one-third of pericardial cysts might get smaller and smaller through time [20].

Kumar et al. reported a giant pericardial cyst in a 5-year-old child with a history of dull, aching chest pain for 3 months. They mentioned that a pericardial cystic structure measuring approximately 10.0 cm × 9.5 cm × 9.0 cm was seen loosely adherent to the pericardium in the surgical procedure. Both the lung fields were clear and the heart chambers were normal in size, but they were compressed posteriorly. For the patient, the postoperative period was without any complication and the child is doing well in follow-up [18].

Hekmat et al. (2016) presented a 24-year-old man referred to the emergency department with dyspnea and persistent cough; no abnormality was found during initial physical medical examination. His medical history was normal. His transthoracic echocardiogram showed an echolucent space next to the right atrium at the right cardio phrenic angle. No pericardial effusion was found. The patient underwent surgery. After midsternotomy, a huge cyst, measuring approximately 13 × 8 × 5 cm^3^ in diameter, was found outside the pericardium on the right side. After 5 days, the patient was discharged, and the pathologic report confirmed the preoperative diagnosis of a pericardial cyst [21].

Blakeslee reported 20 cases of pericardial cysts in children aged lower than 18 [12].

Kumar Kar and Ganguly (2017) review article focused on current methods and procedures for the diagnosis and management of pericardial cysts. They recommended echocardiography for serial follow-up and image-guided aspiration of the pericardial cyst in the presence of compressive effects leading to cardiovascular and airway symptoms. They stated that a systematic approach is desirable for management of pericardial cysts depending on size, shape, and compression effects; symptoms; and easy access to serial echocardiographic follow-up. However, the above tests might fail in differentiating pericardial diverticulum from cysts, and sometimes surgery might be the only solution logical to implement [22].

## 4. Conclusions

This case was a large pericardial cyst in a 9-year-old child by rare presentation, highlighting the fact that the diagnosis of pericardial cysts should be kept in mind in any intrathoracic cystic lesion in children.

## Figures and Tables

**Table 1:** Etiology of pericardial cysts according to previous studies.

Etiology of Pericardial Cyst [18]
1. Congenital
2. Inflammatory: Rheumatic pericarditis, Bacterial infection particularly tuberculosis, Echinococcosis
3. Traumatic
4. Post cardiac surgery
5. Patient on chronic hemodialysis

## Data Availability

The data presented in this study are available on request from the corresponding author from data center of Ali-Ibn-Abitaleb Hospital, Zahedan, Iran. The data are not publicly available due to the observance of citizenship rights and the realization of immunity.
